# Regulation of FN1 degradation by the p62/SQSTM1-dependent autophagy–lysosome pathway in HNSCC

**DOI:** 10.1038/s41368-020-00101-5

**Published:** 2020-12-14

**Authors:** Xinchen Liu, Lin Meng, Xing Li, Daowei Li, Qilin Liu, Yumeng Chen, Xiangwei Li, Wenhuan Bu, Hongchen Sun

**Affiliations:** 1 Department of Oral Biology, Jilin Provincial Key Laboratory of Tooth Development and Bone Remodeling, School and Hospital of Stomatology, Jilin University, Changchun, P.R. China; 2 Department of Endodontics, School and Hospital of Stomatology, Jilin University, Changchun, P.R. China; 3 Department of Pathology, School and Hospital of Stomatology, Jilin University, Changchun, P.R. China; 4 School and Hospital of Stomatology, China Medical University, Shenyang, P.R. China

**Keywords:** Oral cancer detection, Macroautophagy

## Abstract

Epithelial–mesenchymal transition (EMT) is involved in both physiological and pathological processes. EMT plays an essential role in the invasion, migration and metastasis of tumours. Autophagy has been shown to regulate EMT in a variety of cancers but not in head and neck squamous cell carcinoma (HNSCC). Herein, we investigated whether autophagy also regulates EMT in HNSCC. Analyses of clinical data from three public databases revealed that higher expression of fibronectin-1 (FN1) correlated with poorer prognosis and higher tumour pathological grade in HNSCC. Data from SCC-25 cells demonstrated that rapamycin and Earle’s balanced salt solution (EBSS) promoted autophagy, leading to increased FN1 degradation, while 3-methyladenine (3-MA), bafilomycin A1 (Baf A1) and chloroquine (CQ) inhibited autophagy, leading to decreased FN1 degradation. On the other hand, autophagic flux was blocked in BECN1 mutant HNSCC Cal-27 cells, and rapamycin did not promote autophagy in Cal-27 cells; also in addition, FN1 degradation was inhibited. Further, we identified FN1 degradation through the lysosome-dependent degradation pathway using the proteasome inhibitor MG132. Data from immunoprecipitation assays also showed that p62/SQSTM1 participated as an autophagy adapter in the autophagy–lysosome pathway of FN1 degradation. Finally, data from immunoprecipitation assays demonstrated that the interaction between p62 and FN1 was abolished in p62 mutant MCF-7 and A2780 cell lines. These results indicate that autophagy significantly promotes the degradation of FN1. Collectively, our findings clearly suggest that FN1, as a marker of EMT, has adverse effects on HNSCC and elucidate the autophagy–lysosome degradation mechanism of FN1.

## Introduction

EMT is a biochemical process that enables epithelial cells to exhibit traits of a mesenchymal phenotype.^1–3^ Cell surface proteins (E-cadherin, N-cadherin, ZO-1 and Integrin family members), cytoskeletal proteins (α-SMA and Vimentin), extracellular matrix proteins (collagens, FN1 and Laminin) and some transcription factors (PRRX1, SNAIL1, SLUG, TWIST1/2 and ZEB1/2) are biomarkers of EMT (refs. ^4,5^). Previous immunohistochemical analyses of HNSCC samples showed that FN1 is overexpressed in the tumour stromal region and at the invasive front of the tumour.^6^ FN1 is upregulated in many tumours and is then referred to as the cellular or ‘oncofoetal’ variant (OncFN). OncFN is a marker of the tumour vasculature^7^ and a principal component of the metastatic microenvironment, termed the premetastatic niche, in many tumours.^8^ Multivariate analysis showed that overexpression of OncFN was associated with a trend towards significantly lower overall survival rates.^9^ Although the role of FN1 in tumours has been explained, the intracellular metabolism of FN1 and its mechanism are not sufficiently understood. As FN1 is an essential mesenchymal phenotypic indicator, its degradation pathway requires further investigation.

In eukaryotic cells, there are two critical systems for protein degradation: autophagy and the proteasome system.^10^ Autophagy is a critical catabolic process for degrading unwanted cytoplasmic material in the lysosome of the cell.^11,12^ Autophagy performs two primary cellular functions, detoxifying the cell and conferring resistance to nutrient stress by recycling waste, leading to protection against toxic environments and survival under nutrient stress.^13^ In general, the proteasome system has difficulty degrading aggregated proteins, which are usually degraded by selective autophagy. Selective autophagy requires a class of adapter proteins, such as p62/SQSTM1 or an autophagy cargo receptor (e.g., NBR1), to link ubiquitinated proteins and autophagosome membranes.^13,14^

P62 is an autophagy adaptor^10^; it is a multidomain protein that can interact with the autophagy machinery as a key adaptor of target cargo via domains such as Phox1 and Bem1p (PB1) (ref. ^15^) domains, a tumour necrosis factor (TNF)-associated receptor-6 (TRAF6) binding (TB) (ref. ^16^) domain, a ZZ-type zinc finger (ZZ) domain, an LC3-interacting region (LIR), a Keap1-interacting region (KIR) and a ubiquitin-associated domain (UBA) (ref. ^17^). The PB1, LIR and UBA domains are mainly involved in selective autophagy.^18^ P62 tends to aggregate and form discrete puncta known as p62 bodies in cells. The formation of p62 bodies depends on the PB1 domain of p62 (ref. ^15^). Experiments show that oligomers can bind many ubiquitin tags on a single structure. p62 oligomers can form stronger connections with damaged proteins than can a single p62 unit.^19^ The specific role of p62 oligomerization mediated by the PB1 domain in the formation of autophagosomes, however, still needs further exploration. The LC3 interaction region (LIR) is necessary for mammalian protein degradation by autophagy.^20^ P62 interacts with LC3 through the LIR domain to promote the formation of autophagosomes.^21^ The UBA domain of p62 plays an important role in the function of p62. The P62 protein can regulate the interaction between ubiquitinated proteins and autophagosomes through its UBA domain, and autophagosomes are the main controllers of protein degradation through the autophagolysosome pathway.^22^

It has been verified that cytoplasmic p62 expression is increased in oral squamous cell carcinoma (OSCC)^23^ and is associated with poor prognosis.^24^ The function and mechanism of p62, however, have not been fully explored, especially regarding proteins that contribute to cancer progression.

In the current study, we hypothesized that autophagy might play a key role in the regulation of EMT via degradation of FN1 through the p62-dependent autophagy–lysosome pathway in HNSCC. Interestingly, our results supported our hypothesis.

## Results

### Characteristics of HNSCC

**Fig. 1:**
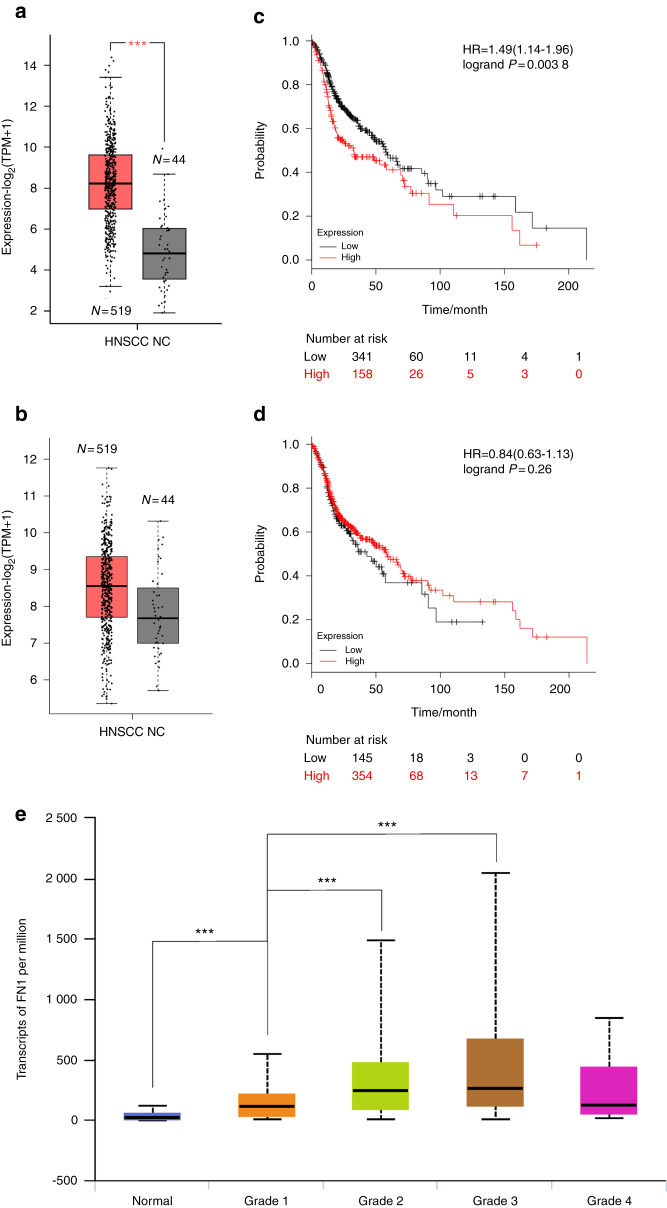
Characteristics of HNSCC in public databases. **a** FN1 expression profile in normal and HNSCC tissue. **b** Vimentin expression profile in normal and HNSCC tissue. **c** The overall survival rates of HNSCC patients with high and low FN1 expression levels. **d** The overall survival rates of HNSCC patients with high and low Vimentin expression levels. **e** Association between HNSCC pathological grade and FN1 expression. NC, normal control tissue. ***P < 0.001

A fundamental change during EMT is the loss of E-cadherin, and the next phase is the activation of Vimentin and expression of FN1 (ref. ^1^). To understand the relationship between FN1/vimentin and HNSCC, we first used three publicly available databases to analyse clinical-related associations between FN1/vimentin and HNSCC. Analysis of data from the GEPIA (ref. ^25^) database (Fig. 1a) showed that Vimentin expression was higher in HNSCC samples than in normal controls (*n* = 44), although the difference was not significant (Fig. 1b). Analysis of data from the Kaplan–Meier Plotter database^26^ showed that the overall survival rate of HNSCC patients (*n* = 519) with high expression of FN1 (*n* = 158) was significantly lower than that of HNSCC patients with low expression of FN1 (*n* = 341) (Fig. 1c). Unexpectedly, the overall survival rate of HNSCC patients (*n* = 519) with high expression of Vimentin (*n* = 354), the most common mesenchymal-related marker, was not different from that of HNSCC patients with low expression of Vimentin (*n* = 145) (Fig. 1d). Interestingly, analysis of data from the UALCAN database^27^ further showed that the expression of FN1 increased with an increase in pathological grade from grade 0 to grade 3 and appeared to plateau at grade 4 (Fig. 1e). These analyses indicate that FN1 has a close association with pathological changes in HNSCC patients.

### Autophagic activity in different OSCC cell lines

**Fig. 2:**
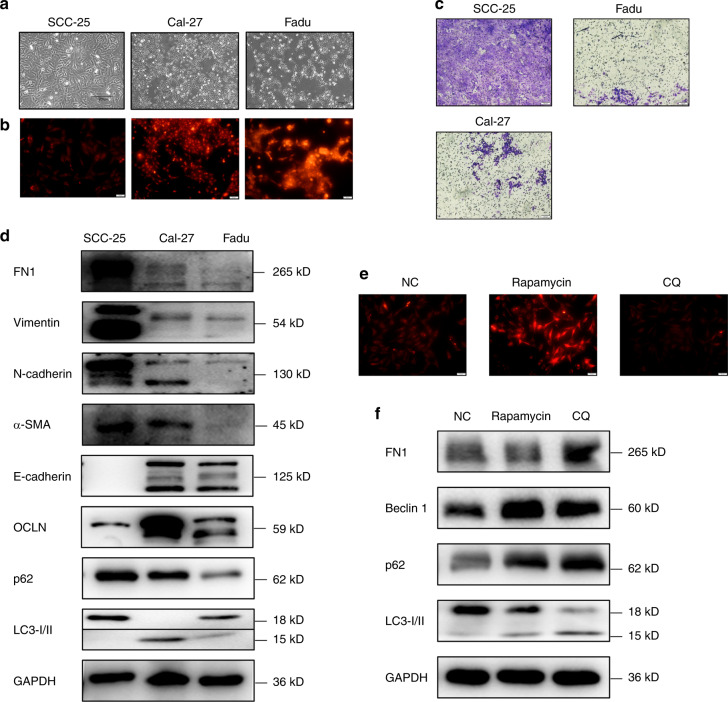
Levels of EMT-related proteins and autophagy-related proteins in SCC-25, Cal-27 and Fadu cells. **a** General morphology of cultured SCC-25, Cal-27 and Fadu cells. **b** AO staining data. **c** Migration assay data. **d** Western blot data. **e** AO staining data. **f** Western blot data. Western blot analysis was performed to detect the expression of FN1 and autophagy-related markers (p62, beclin1, LC3B-I/II) in SCC-25 cells stimulated with rapamycin (200 nmol·L^−1^) or CQ (10 μg·mL^−1^) for 24 h. Experiments were repeated twice

The above analyses clearly demonstrate that FN1 and vimentin, especially FN1, have a close association with HNSCC. To further understand EMT in OSCC and evaluate whether EMT is associated with autophagy in OSCC, three OSCC cell lines, SCC-25, Cal-27 and Fadu, were used in experiments in vitro. SCC-25 cells exhibited a polygonal morphology, Cal-27 cells exhibited a natural diamond-shaped morphology, and Fadu cells were rhomboid with slightly smaller angles than the other two cell lines (Fig. 2a). Next, we evaluated N-cadherin, Vimentin, FN1, α-SMA, E-cadherin and OCLN protein levels in SCC-25, Cal-27 and Fadu cells by western blotting. The data showed that E-cadherin/OCLN protein expression was lower and Vimentin/FN1/N-cadherin/α-SMA protein expression was higher in SCC-25 cells, than in Cal-27 and Fadu cells (Fig. 2d). During autophagy, cytoplasmic LC3-I (microtubule-associated protein light chain 3) is conjugated to phosphatidylethanolamine to form an LC3-phosphatidylethanolamine conjugate (LC3-II), which is located on autophagosome membranes. The expression of P62, an adapter for ubiquitinated connexin proteins and LC3-II (ref. ^28^), was increased. Interestingly, only LC3-I, with higher expression of the p62 protein, was found in SCC-25 cells, while LC3-II, with lower expression of p62, was found in Cal-27 and Fadu cells (Fig. 2d). These data indicated that among these three OSCC cell lines, SCC-25 exhibited characteristics that were most similar to the phenotype of mesenchymal cells, with higher expression of FN1 and lower autophagic activity, and that both Cal-27 and Fadu cells showed characteristics that were more similar to the phenotype of epithelial cells, with higher autophagic activity and low FN1 expression, suggesting that EMT OSCC is indeed associated with autophagy. We also used acridine orange (AO) staining to evaluate autophagolysosomes. The results showed that Fadu and Cal-27 cells had a higher number of autophagolysosomes than SCC-25 cells. These results indicated that Fadu and Cal-27 cells had much higher autophagic activity than SCC-25 cells (Fig. 2b). Further migration assays demonstrated that after EMT, SCC-25 cells had a greatly enhanced migration ability compared to Cal-27 and Fadu cells (Fig. 2c). Therefore, in subsequent investigations, we only focused on SCC-25 cells as an in vitro model to understand autophagy and EMT in OSCC. Rapamycin and CQ are a classical autophagy activator and inhibitor, respectively. Figures 2e and f show that rapamycin also stimulated autophagy, with a decrease in FN1 expression, while CQ inhibited autophagosomal degradation, with an increase in FN1 expression. These results suggest that autophagy is involved in the regulation of the EMT-related protein FN1.

### Effects of autophagy on the degradation of FN1

Detection of LC3-II and p62 levels can be used as an indirect method to determine the presence of autophagosomes. The protein content of LC3-II and p62 can indicate the number of autophagosomes. When autophagy was enhanced, autophagosome formation exceeded degradation, and the levels of LC3-II and p62 increased; when autophagy was impaired, autophagosome formation was lower than degradation, and the levels of LC3-II and p62 decreased. Unfortunately, there is not always a clear positive correlation between the impairment of autophagy or autophagic flux and the increase in LC3-II or the decrease in p62 levels. Therefore, analysis of p62 can only help assess the impairment of autophagy or autophagic flux. The specific condition of autophagic flux needs to be observed in combination with multiple indicators.^28^

**Fig. 3:**
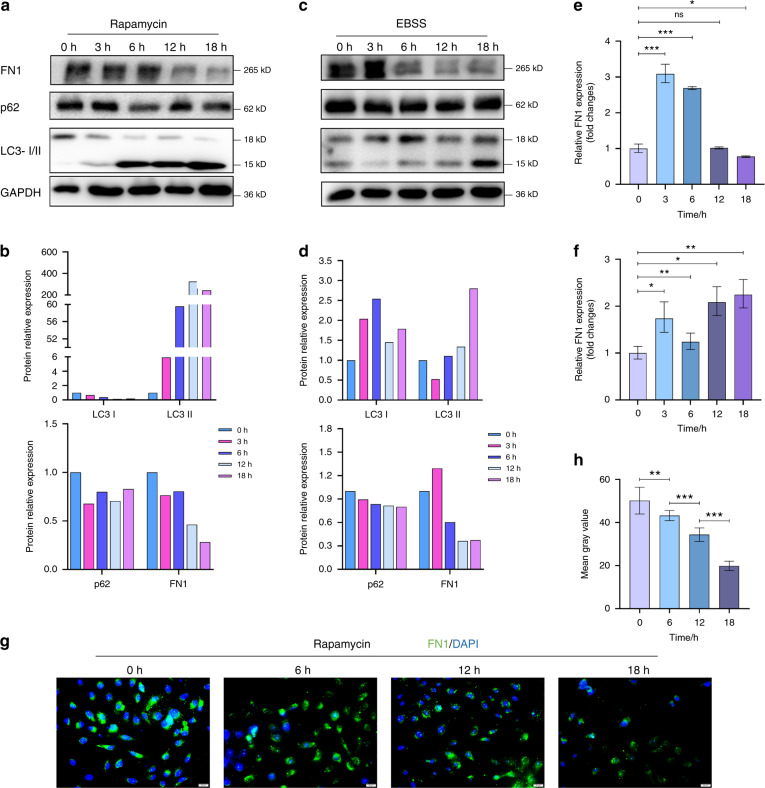
Effects of autophagy activators on autophagy and FN1 expression in SCC-25 cells. **a** Western blot data for cells treated with the activator rapamycin (200 nmol·L^−1^). **b** Bar graphs of the results in (**a**); the intensity of each protein band was normalized to that of GAPDH. **c** Western blot data for cells treated with the activator EBSS. **d** Bar graphs of the results in (**c**); the intensity of each protein band was normalized to that of GAPDH. **e** RT-qPCR data of FN1 expression in cells treated with the activator rapamycin (200 nmol·L^−^^1^) or EBSS (**f**). **g** FN1 expression, as detected by immunofluorescence, in cells treated with the activator rapamycin. **h** Quantification of the average fluorescence values of single cells in (**g**). Experiments were repeated twice

To further understand the connections between FN1 and autophagy, we dynamically examined changes in the expression of FN1 and autophagic activity using two kinds of autophagy activators, rapamycin and EBSS. P62, LC3-I and LC3-II were used as markers of autophagic activity. In general, both autophagy activators resulted in similar trends, gradually increasing the conversion of LC3-I to LC3-II, while the protein level of FN1 gradually decreased over time up to 18 h (Figs. 3a–d, g and h). Under induction by rapamycin and EBSS, the level of p62 was slightly decreased. These results indicated that the total number of mature autophagosomes was reduced (Figs. 3a–d). Moreover, the mRNA level of FN1 was inversely related to the protein level, regardless of whether rapamycin or EBSS was used to induce autophagy (Figs. 3e and f). Immunofluorescence staining of the rapamycin group demonstrated that the autophagy activator rapamycin decreased the protein level of FN1 in SCC-25 cells (Figs. 3g and h) and SCC-15 cells (Supplement Fig. 1a). These data indicate that the increased autophagic activity in SCC-25 cells can decrease the FN1 protein level.

**Fig. 4:**
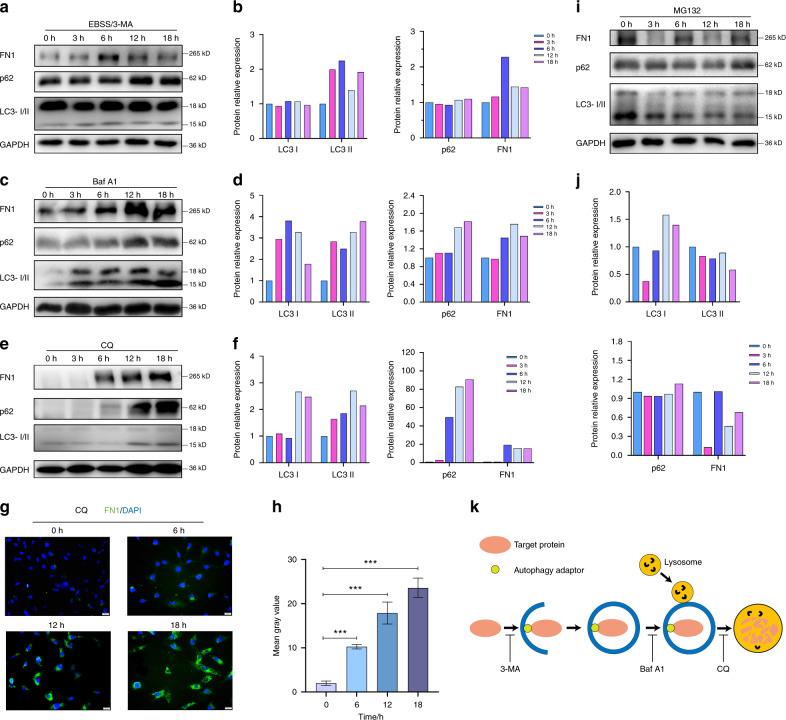
Effects of autophagy inhibitors and proteasome inhibitors on autophagy and FN1 expression in SCC-25 cells. **a** Western blot data from cells treated with the inhibitor 3-MA (500 μmol·L^−1^) with starvation. **b** Bar graphs of the results in (**a**). **c** Western blot data from cells treated with the inhibitor Baf A1 (500 nmolL·L^−1^). **d** Bar graphs of the results in (**c**). **e** Western blot data from cells treated with the inhibitor CQ (10 μg·mL^−1^). **f** Bar graphs of the results in (**e**). **g** FN1 expression, as detected by immunofluorescence, in cells treated with the inhibitor CQ. **h** Quantification of the average fluorescence values of single cells in (**g**). **i** Western blot data from cells treated with the proteasome inhibitor MG132 (50 µmol·L^−^^1^). **j** Bar graphs of the results in (**i**). For all bar graphs, the intensity of each protein band was normalized to that of GAPDH. **k** Chart of protein degradation through the autophagy–lysosome pathway and the site of action of 3-MA, Baf-A1 or CQ. Experiments were repeated twice

Next, we sought to understand whether the autophagy–lysosome pathway is the only pathway for FN1 degradation. Therefore, we tried to block autophagy at different stages. As Fig. 4k indicates, when autophagy was blocked at different stages, the change trends LC3 and p62 were different. It needs to be emphasized that when the formation of autophagosomes is blocked, lysosomes still function.^29^ Although LC3-I cannot be transformed into LC3-II, residual autophagosomes can still be degraded by lysosomes.^30^

In the first experiment, the data shown in Fig. 4a confirmed that 3-methyladenine (3-MA), when used under starvation conditions (EBSS), inhibits the formation of autophagosome precursors.^31^ These data showed that the levels of proteins related to autophagy formation, LC3-II/I and p62, remained basically unchanged. The inhibitory effects of 3-MA/EBSS on autophagy (Fig. 4k) did not block lysosomal function, and LC3-II was still degraded (Fig. 4a). Our data demonstrated that inhibition of autophagy indeed caused the deposition of FN1 (Figs. 4a and b). In the second experiment, Baf A1, which inhibits lysosomal function by inhibiting vacuolar-type H+-ATPase (V-ATPase) and P-type ATPases,^32^ was used to block the fusion of the autophagosome with the lysosome (Fig. 4k). The data in Figs. 4c and d show that p62 and LC3-II accumulated in the cells. In addition, the process of FN1 degradation was hindered. In the third experiment, CQ was used. CQ acts as a lysosomal lumen alkalizer, preventing the final step of degradation in the autolysosome (Fig. 4k).^33^ The data showed that CQ strongly inhibited autophagic activity and blocked the degradation of FN1 in SCC-25 cells (Figs. 4e, f, g and h) and Baf A1 strongly blocked the degradation of FN1 in SCC-15 cells (Supplement Fig. 1b) until 18 h. Autophagy and the proteasome system are two critical systems for protein degradation in cells. Therefore, in the next experiment, it was necessary to determine whether the proteasome system is also involved in degradation. A proteasome inhibitor, MG132, affected the level of FN1 but did not cause the aggregation of FN1 in SCC-25 cells (Figs. 4i and j). Collectively, the data from these four experiments clearly demonstrate that FN1 degradation is not related to the ubiquitin–proteasome degradation system and that blockade of autophagy results in the accumulation of FN1, which indicates that autophagy is involved in the degradation of FN1.

To further verify whether autophagy is involved in the process of FN1 degradation, we used the BECN1 mutant HNSCC cell line (Supplement Table) and Cal-27 cells. The results showed that rapamycin and EBSS did not promote the degradation of p62 and FN1 or the conversion of LC3-I to LC3-II in Cal-27 cells (Supplement Fig. 2a-d). These results indicate that autophagy defects can inhibit the degradation of FN1.

### Roles of p62 in FN1 degradation

**Fig. 5:**
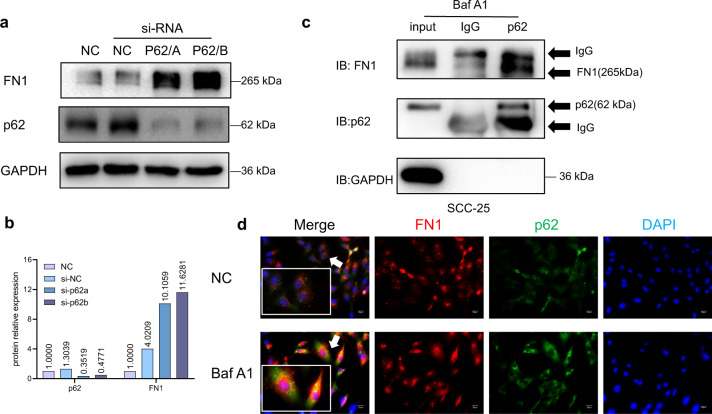
Role of p62 in the degradation of FN1 in SCC-25 cells. **a** Western blot data from p62 siRNA assay. **b** Bar graphs of the results in (**a**); the intensity of each protein band was normalized to that of GAPDH. **c** Western blot data from the immunoprecipitation assay. SCC-25 cells were treated with Baf A1 (500 nmol·L^−1^) for 12 h, and immunoprecipitation was then performed with an anti-p62 antibody. **d** FN1 and p62 immunofluorescence colocalization assay. Experiments were repeated twice

**Fig. 6:**
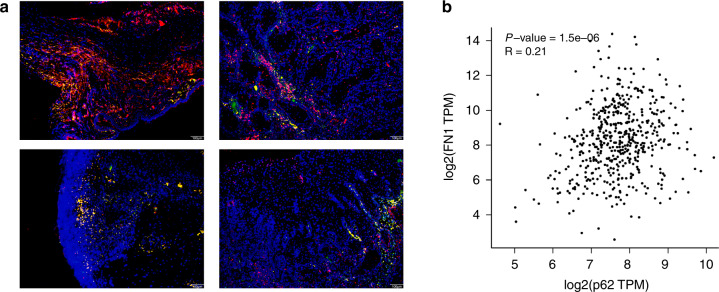
Relation between p62 and FN1 in HNSCC patients. **a** FN1 and p62 immunofluorescence colocalization assay in human HNSCC tissue. Tissues were randomly selected from 10 patients with HNSCC. **b** Correlation analysis of FN1 and p62 mRNA levels in HNSCC samples from the TCGA database using GEPIA

Interestingly, a correlation between the levels of p62 protein and FN1 was observed in the above experiments, which led us to uncover the relationship between p62 and FN1/EMT. It is known that protein degradation through the autophagy pathway requires adapter proteins. The above data confirmed that FN1 is degraded through the autophagy–lysosome pathway but did not confirm whether p62 is the adapter between FN1 and the autophagosome membrane. P62 siRNA experiments showed that FN1 accumulated after the expression of p62 was blocked (Figs. 5a and b). In addition, an immunoprecipitation assay with an anti-p62 antibody showed that the anti-p62 antibody pulled down the FN1 protein (Fig. 5c). Next, immunofluorescence staining demonstrated that FN1 and p62 were colocalized in the cells (Fig. 5d). Analysis of data from GEPIA also showed that the level of p62 was significantly correlated with that of FN1 (Fig. 6b). Finally, we verified the distinct correlation between p62 and FN1 in tissue sections from OSCC patients (Fig. 6a). Moreover, the p62 protein was mainly localized in the cytoplasm, and the phenomenon of colocalization with FN1 indicated that oncofoetal FN1 was indeed present in the cytoplasm of cancer cells (Fig. 6a). These data suggest that p62 is an adapter between FN1 and the autophagosome membrane, is closely related to FN1 degradation in cells and is an important protein in the regulation of autophagy and EMT in OSCC.

### Roles of the PB1 and UBA domains of p62 in the degradation of FN1

**Fig. 7:**
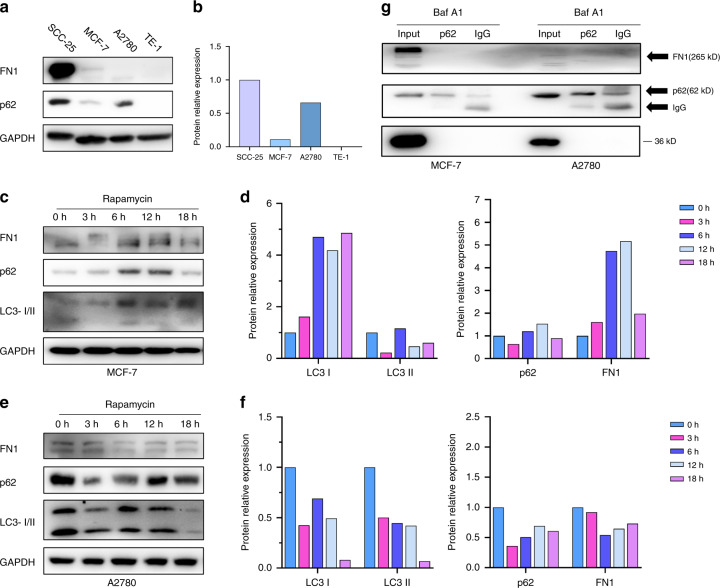
Role of the PB1 and UBA domains of p62 in the degradation of FN1. **a** Western blot data from SCC-25, MCF-7, A2780 and TE-1 cells. **b** Bar graphs of the results in (**a**). **c** Western blot data from MCF-7 cells treated with the activator rapamycin (200 nmol·L^−1^). **d** Bar graphs of the results in (**c**). **e** Western blot data from A2780 cells treated with the activator rapamycin (200 nmol·L^−1^). **f** Bar graphs of the results in (**e**). For all bar graphs, the intensity of each protein band was normalized to that of GAPDH. **g** Western blot data from the immunoprecipitation assay. Both MCF-7 and A2780 cells were treated with Baf A1 (500 nmol·L^−1^) for 12 h, and immunoprecipitation was then performed with an anti-p62 antibody. Experiments were repeated twice

To understand the roles of the PB1 and UBA domains of p62 in the degradation of FN1, the expression of p62 and FN1 was evaluated in TE-1, A2780, MCF-7 and SCC-25 cells. The results demonstrated that the expression of both p62 and FN1 was significantly decreased or very difficult to detect in TE-1 and MCF-7 cells compared to SCC-25 cells (Figs. 7a and b). Interestingly, the expression of p62 in A2780 cells was only slightly decreased compared to that in SCC-25 cells, although the expression of FN1 was significantly decreased (Figs. 7a and b). Further, the expression of FN1 increased in p62-mutated MCF-7 (Supplement Table) cells during rapamycin induction (Figs. 7c and d). The expression of FN1 did not decrease significantly in p62-mutated A2780 (Supplement Table) cells under rapamycin induction (Figs. 7e and f). To reveal the role of the PB1 domain and UBA domain in selective autophagy, we performed immunoprecipitation assays. The results showed that mutation of PB1 (MCF-7) and UBA (A2780) resulted in failure of the interaction between p62 and FN1 (Fig. 7g). These results reveal that the PB1 domain and UBA domain indeed play an important role in selective autophagy. Our data also demonstrated that Baf A1 can still inhibit lysosomal acidification and autophagosomal degradation in cells with BECN1 or p62 mutations, leading to impaired degradation of FN1 (Supplement Figs. 2e, f and 3a-d). These results indicate that the lysosome is the major site of FN1 degradation, although we still do not know how FN1 enters the lysosome in cells with autophagic flux defects or autophagic ligand defects.

## Discussion

EMT is a critical process during development, tissue repair and acquisition of the invasive phenotype of cancer. In this study, we recognized that FN1 is an important marker of EMT and is degraded via the autophagy–lysosome pathway. p62 acts as an autophagy adapter protein to link FN1 to autophagosomes, resulting in intracellular degradation of FN1. Analyses of clinical data from three public databases indicated that FN1 has a strong association with pathological grade and survival rate in HNSCC. Therefore, autophagy can regulate EMT to affect the cancer phenotype in HNSCC.

As a consequence of EMT, epithelial cells gradually lose their hallmarks of high differentiation and gain a certain degree of characteristics associated with redifferentiation, including migratory, invasive, metastatic, and stem cell characteristics as well as apoptosis resistance.^3,4,34,35^ Accordingly, after interstitialization of cancer cells, patients are more likely to experience distant metastasis, recurrence, and radiochemotherapeutic resistance.^3,4,34,35^ Several studies have reported a direct or indirect effect of autophagy on EMT regulation in various diseases.^36–38^ In age-related macular degeneration (AMD), retinal pigment epithelium (RPE) can stabilize keratin8 (KRT8) expression via autophagy, therefore inhibiting EMT and protecting RPE cells.^38^ Autophagy inhibition explicitly promotes EMT and invasion in RAS-mutated cancer cells.^36^ In squamous cell carcinoma, autophagy deficiency promotes cell proliferation and migration through p62-dependent stabilization of the EMT transcription factor Twist1 (ref. ^37^). Collectively, these observations indicate that autophagy can regulate the expression of EMT-related markers. Clinical data from three public domain databases suggest that FN1 has a strong association with pathological grade and survival rate in HNSCC (Fig. 1). A higher level of FN1 expression was significantly associated with a higher pathological grade and a lower survival rate (Fig. 1). In vitro data indicated that higher autophagic activity decreased the protein level of FN1, while inhibition of autophagy promoted an increase in the protein level of FN1 (Figs. 2 and 4; Supplement Figs. 1b, 2e, 2f and 3c). FN1 is overexpressed in the tumour stroma and at the invasive front in HNSCC (ref. ^6^). FN1 is a marker of the tumour vasculature in cancer cells^7^ and is a principal component of the metastatic microenvironment, termed the premetastatic niche, in many kinds of tumours.^8^ FN1 is a critical protein in EMT and plays an essential role in tumour invasion and metastasis in HNSCC.

Vimentin has been regarded as a marker of metastasis in a variety of cancers, such as breast cancer^39^ and non-small cell lung carcinoma.^40^ In triple-negative breast cancer (TNBC), Vimentin is regarded as an indicator of poor prognosis.^39^ In non-small cell lung carcinomas, high expression of Vimentin is thought to be associated with metastatic events.^40^ However, analysis of clinical data from three public databases revealed that increased expression of Vimentin was not associated with overall survival in HNSCC patients (Figs. 1b and d). Therefore, it is interesting to understand the roles of FN1 in HNSCC.

Autophagy and the proteasome system are two critical systems for protein degradation in eukaryotic cells.^10^ Some of the degradation pathways of EMT-related protein markers have been explored clearly. For instance, twist is degraded by both the autophagy and proteasome pathways.^37^ Snail was found to be degraded naturally through autophagy in a different model.^41–43^ Vimentin has been identified to be ligated to the autophagy adapter protein p62, which is presumably associated with autophagy pathways.^44^ However, its misfolded body is degraded by the proteasome system.^45^ FN1 in the cytoplasm of tumour cells is called oncofoetal fibronectin (OncFN). OncFN plays an essential role in HNSCC and EMT, as clearly stated above. Consequently, it is imperative to conduct an intensive study of the intracellular degradation of FN1. Our results indicated that although the amount of p62 protein did not decrease significantly under autophagy activation, the accelerated autophagic flux resulted in rapid degradation of FN1 (Fig. 3). In some cases, autophagy activation cannot lead to a significant decrease in p62. This phenomenon may be related to cell heterogeneity.^28^ In some cell types, despite the strong level of autophagy induction, the total amount of p62 did not change. This stability was affected by the tandem MRFP/mCherry-GFP-LC3 reporter and the turnover of ATG7 and lysosome-dependent cargo protein (C.T. Chu, personal observation). In other cases, the robust loss of p62 was not related to the increase in autophagic flux as assessed by luciferase-based flux measurements.^46^ p62 may be transcriptionally upregulated under certain conditions, further complicating the interpretation of the results.^47–50^ In some situations, the degradation of p62 may be delayed compared with the increase in LC3-II, and sometimes it must be observed for 48 h.^28^ Correspondingly, blocking autophagic flux results in degradation of FN1 (Fig. 4). Furthermore, the results of lysosomal inhibitor and proteasome inhibitor assays demonstrated that FN1 was ultimately degraded in organelles such as lysosomes rather than in proteasomes (Fig. 4). Autophagy adaptors are critical bridges for protein degradation dependent on the autophagy–lysosome pathway. They correctly recognize cargo and tether it tightly to the nascent autophagosome membrane.^10^ Our results revealed that p62 is the autophagy adaptor connecting FN1 with the autophagosome membrane and that p62 and FN1 are colocalized in OSCC cells (Figs. 5, 6 and 7).

In tumour cells, autophagy is regarded as a double-edged sword. Currently, many drugs related to the regulation of autophagy have been put into clinical use. It has been shown that the level of autophagy in cancer cells is higher than that in normal tissues, and conventional radiotherapy and chemotherapy can induce protective autophagy in cancer cells. Therefore, directly targeting autophagy is a therapeutic strategy for cancer.^51^ CQ, Baf A1 and epigallocatechin gallate (EGCG) (ref. ^52^) are typical representatives. However, the therapeutic strategy of inhibiting autophagy can induce tumour radiotherapeutic/chemotherapeutic resistance and metastasis.^51^ According to our research, the above malignant behaviours may be related to EMT. Metformin is a classic blood glucose regulator and an autophagy inducer. It has been shown that metformin can block tumour metastasis by inhibiting tumour cell EMT (ref. ^53^). Therefore, taking EMT-related proteins as a starting point to explore the precise role of activated autophagy in tumour therapy will benefit tumour therapy.

In conclusion, our data indicate that FN1 is degraded by the p62-dependent autophagy–lysosome pathway, independent of the ubiquitin–proteasome pathway, and indicate that FN1 is a potential prognostic biomarker for HNSCC because it has a strong association with EMT and tumour invasion and metastasis (Supplement Fig. 4). In the future, we aim to investigate whether we can inhibit the expression of FN1 by interfering with autophagy to control the progression of cancer.

## Methods

### Cell cultures

Cal-27 cells, a human oral squamous cell carcinoma (OSCC) line with a key mutation in the autophagy protein BECN1 (Supplement Table), were kindly provided by Dr Shi Songtao, Shanghai Ninth People’s Hospital, China. The following cell lines were obtained from ATCC (Rockville, MD, USA): SCC-25, SCC-15 and Fadu (human OSCC cell lines); A2780 (a human ovarian cancer cell line); TE-1 (a human oesophageal cancer cell line); and MCF-7 (a human breast cancer cell line). A2780, TE-1 and MCF-7 cells have mutations in p62 (Supplement Table) (ref. ^33^). The mutation site in MCF-7 cells is located in the PB1 domain, and the mutation site in A2780 cells is located in the UBA domain (Supplement Table). Cal-27, SCC-25 and Fadu cells were cultured in H-DMEM (Bioind, Kibbutz Beit HaEmek, Israel). A2780, MCF-7 and TE-1 cells were cultured in RPMI-1640 (Bioind, Kibbutz Beit HaEmek, Israel). SCC-15 cells were cultured in Ham’s F12 medium (HyClone, Utah, USA). All media were supplemented with 10% foetal bovine serum (FBS) (Bioind, Guangzhou, Guangdong, China) and 1% penicillin/streptomycin (HyClone). During starvation induction, cells were cultured in Earle’s balanced salt solution (EBSS) (HyClone). All cells were incubated in a thermostatic incubator (SANYO, Osaka, Japan) at 37 °C in 5% CO_2_.

### Autophagy induction and inhibition assays

Rapamycin (200 nmol·L^−1^), 3-MA (500 μmol·L^−1^) under nutrient-rich conditions, or Earle’s balanced salt solution (EBSS) was used to activate autophagy and simulate nutritional deficiency status (starvation status). 3-MA (500 μmol·L^−1^) with starvation, bafilomycin A1 (500 nmol·L^−1^), or chloroquine (CQ, 10 μg·mL^−1^) was used to inhibit autophagy. We used EBSS to induce starvation status and normal medium as a nutrient-replete condition.

### Migration experiment

The migration ability was measured based on the number of cells that migrated across the Transwell filter (8-μm pores, Guangzhou, China). Cells were cultured in serum-free H-DMEM in the upper chambers. H-DMEM containing 20% FBS was added to the lower chambers. After cells were cultured for 24 h, cells that had migrated to the opposite side of the Transwell filter were stained with crystal violet staining solution (Beyotime, Shanghai, China).

### Western blot analysis

Samples were lysed in RIPA buffer supplemented with protease inhibitors (Santa Cruz, Texas, USA) at 4 °C. The protein concentration was determined using a BCA Protein Assay Kit (Beyotime, Shanghai, China). Fifty micrograms of protein from each sample was separated on 8%–10% SDS-PAGE gels and subsequently transferred to a 0.45 µm pore size PVDF membrane (GE Healthcare Life Sciences, Massachusetts, USA). After blocking at room temperature for 2 h, the membrane was incubated with anti-E-cadherin, anti-Vimentin, anti-p62, anti-LC3B, anti-Beclin1, anti-GAPDH and anti-FN1 primary antibodies (Proteintech Group, Rosemont, PA, USA) at 4 °C overnight. After incubation with a HRP-conjugated secondary antibody for 2 h at room temperature, HRP signals were detected using a hypersensitive ECL Chemiluminescence Detection Kit (Proteintech Group). The results were analysed using the densitometric analysis software Quantity One v4.6.2 for Windows (Bio‑Rad, CA, USA). GAPDH was used as the loading control.

### Immunofluorescence assays

Ten primary OSCC specimens were obtained from the Department of Oral Pathology and Department of Oral Maxillofacial Surgery, Hospital of Stomatology, Jilin University. The protocol was approved by the Ethics Committee of Jilin University. Tissues were fixed in 10% formaldehyde solution, embedded in paraffin and cut into 4-μm-thick sections. Antigen retrieval was performed by microwave heating in Tris-EDTA buffer (pH = 9) and blocking with 5% BSA-PBS.

Cells were grown on glass cover slips, fixed with anhydrous ethanol at room temperature for 20 min, permeabilized in 0.3% Triton X-100 for 10 min, and blocked with 5% BSA-PBS at 37 °C for 2 h.

Then, both human tissue sections and cells on glass cover slips were incubated with an anti-FN1 antibody (1:200 dilution) at 4 °C for 12 h. Human tissue sections were stained with a Cy3-conjugated antibody, and cells on glass cover slips were labelled with a FITC-conjugated secondary antibody at 4 °C for 12 h. Next, all stained samples were incubated with an anti-p62 antibody (1:200 dilution) at 4 °C for 12 h. For immunofluorescence colocalization assays, a second incubation with the primary antibody was performed. Finally, all stained samples were stained with DAPI for 2 min. The mean grey values were semiquantitatively analysed with ImageJ.

### Real-time quantitative polymerase chain reaction (RT-qPCR)

Total RNA was extracted from cells with TRIzol reagent (Thermo Fisher Scientific, California, USA), and 1 μg of total mRNA was used to synthesize cDNA using 5× PrimeScript RT Master Mix (TaKaRa, Da Lian, China). qPCR was performed using SYBR Premix Ex Taq (TaKaRa). The relative mRNA expression levels were normalized to those of the internal control β-actin. The sequences of the primers are listed below. FN1, ACAGAACTATGATGCCGACCAGAAG and CTGATCTCCAATGCGGTACATGA; β-actin, GGAGATTACTGCCCTGGCTCCTA and GACTCATCGTACTCCTGCTTGCTG.

### siRNA transfection

Two siRNAs targeting p62A (5′-CCUACGUGAAGGAUGACAUTT-3′) and p62B (5′-CCAGACUACGACUUGUGUATT-3′) and a control siRNA (5′-UUCUCCGAACGUGUCACGUTT-3′) were obtained from GenePharma Co., Ltd (Shanghai, China). siRNAs were transfected into cells using Lipofectamine 2000 Transfection Reagent (Thermo).

### Co-immunoprecipitation assays

First, SCC-25 cells were incubated with Baf A1 (company name, city, state, country) for 12 h, washed with ice-cold PBS three times and lysed with IP lysis buffer (Proteintech, KIP-1) containing a protease inhibitor. Cell lysates were centrifuged at 2 500 r·min^−1^ for 5 min. Supernatants were collected and incubated with an anti-p62 antibody or rabbit IgG overnight at 4 °C. Protein agarose beads were mixed at room temperature for 2 h and were then centrifuged to obtain the precipitate. The beads were washed with PBS four times. Finally, the precipitated proteins were eluted and denatured in 5× SDS loading buffer and analysed by western blotting.

### Acridine orange (AO) staining

To quantify the number of cells with acidic vesicles, cells were stained with 1 μg/mL AO in PBS at 37 °C for 15 min. After incubation, cells were washed with PBS.

### Statistical analysis

All data are presented as the mean ± SD of three independent experiments. Data were analysed using SPSS (version 22.0). A *P*-value of < 0.05 was considered significant.

## Supplementary information

Supplement Figure 1

Supplement Figure 2

Supplement Figure 3

Supplement Figure 4

Supplement Table
