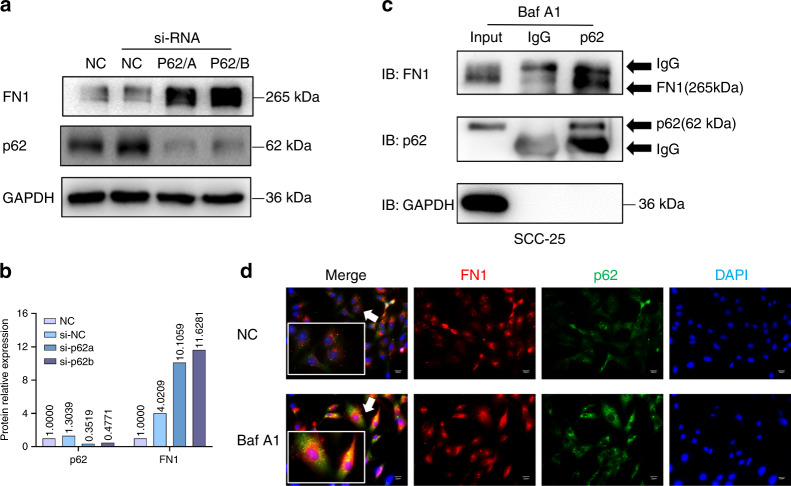# Correction: Regulation of FN1 degradation by p62/SQSTM1-dependent autophagy–lysosomal pathway in HNSCC

**DOI:** 10.1038/s41368-021-00139-z

**Published:** 2021-11-02

**Authors:** Xinchen Liu, Lin Meng, Xing Li, Daowei Li, Qilin Liu, Yumeng Chen, Xiangwei Li, Wenhuan Bu, Hongchen Sun

**Affiliations:** 1 Department of Oral Biology, Jilin Provincial Key Laboratory of Tooth Development and Bone Remodeling, School and Hospital of Stomatology, Jilin University, Changchun, P.R. China; 2 Department of Endodontics, School and Hospital of Stomatology, Jilin University, Changchun, P.R. China; 3 Department of Pathology, School and Hospital of Stomatology, Jilin University, Changchun, P.R. China; 4 School and Hospital of Stomatology, China Medical University, Shenyang, P.R. China

**Keywords:** Oral cancer detection, Macroautophagy

Correction to: *International Journal of Oral Science* 10.1038/s41368-020-00101-5, published online 14 December 2020

Following publication of this article,^1^ the authors reported the below errors.

**1. Reason for modification: ambiguous description**

Third section of RESULTS: Effects of autophagy on the degradation of FN1

Lines 17–20, paragraph 4: The data showed that CQ strongly inhibited autophagic activity and blocked the degradation of FN1 in SCC-25 cells (Figs. 4e, f, g and h) and SCC-15 cells (Supplement Fig. 1b) until 18 h.

Corrected to: The data showed that CQ strongly inhibited autophagic activity and blocked the degradation of FN1 in SCC-25 cells (Figs. 4e, f, g and h) and **Baf A1 strongly blocked the degradation of FN1 in** SCC-15 cells (Supplement Fig. 1b) until 18 h.

DISCUSSION:

The first sentence of paragraph 3: Vimentin has been excluded as a marker of metastasis in a variety of cancers, such as breast cancer and non-small cell lung carcinoma.

Corrected to: Vimentin has been **regarded** as a marker of metastasis in a variety of cancers, such as breast cancer and non-small cell lung carcinoma.

**2. Reason for modification: Misaligned with the content of Figure**

Second section of RESULTS: Autophagic activity in different OSCC cell lines

Line 14: (Fig. 2c).

Corrected to: (Fig. 2d).

Third section of RESULTS: Effects of autophagy on the degradation of FN1

Line 1, paragraph 4: Fig. 4k

Corrected to: Fig. 4a

The last section of RESULTS: Roles of the PB1 and UBA domains of p62 in the degradation of FN1

The fourth line from the bottom: (Supplement Figs. 2c, 2d and 3a-d).

Corrected to: (Supplement Figs. 2e, f and 3a-d).

**3. Reason for modification: Mistakes of figure legend serial number sequence**

Legend of Fig. 2:

Line 2: **c** Western blot data. **d** Migration assay data. **e** Western blot data.

Corrected to: **c** Migration assay data. **d** Western blot data. **e** AO staining data. **f** Western blot data.

Legend of Fig. 4:

Line 5: **h** Quantification of the average fluorescence values of single cells in Fig. 3g.

Corrected to: **h** Quantification of the average fluorescence values of single cells in (**g**).

Fig. 5:

b, c serial number error, b label content is incorrect.

Original Figure 5:
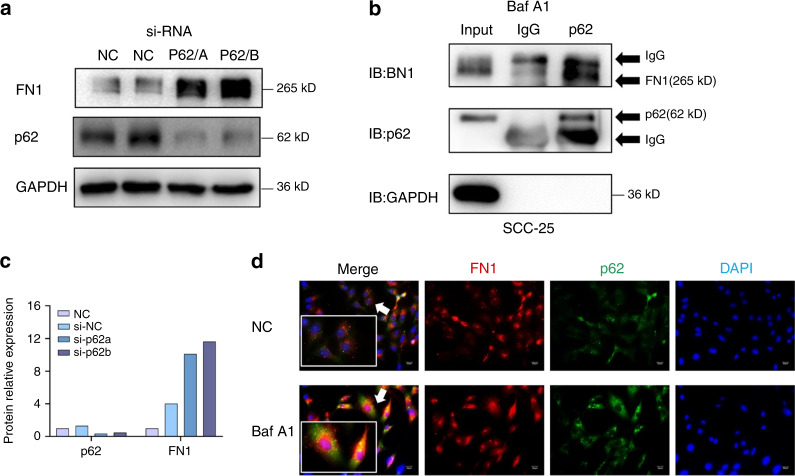


Corrected to: Switch the serial numbers b and c. IB: BN1 changed to IB: FN1.

Revised Figure 5: