# Supplementary figures and images for: Regulation of FN1 degradation by the p62/SQSTM1-dependent autophagy–lysosome pathway in HNSCC
Source: Int J Oral Sci. 2020 Dec 14;12:34. doi: 10.1038/s41368-020-00101-5 (PMC7736930; doi:10.1038/s41368-020-00101-5)

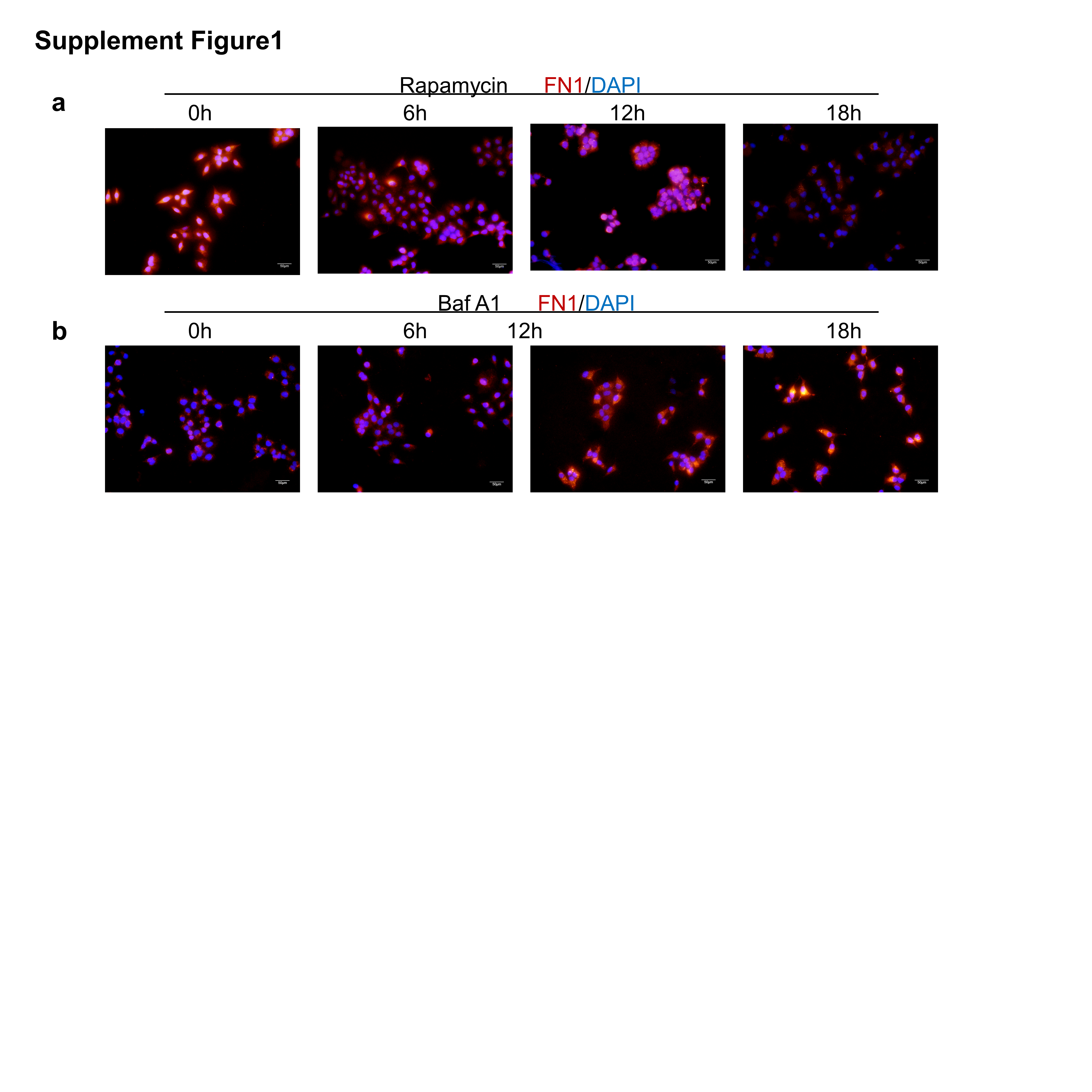

Supplement: Supplementary file 1 — Supplement Figure 1 [file 41368_2020_101_MOESM1_ESM.tif]

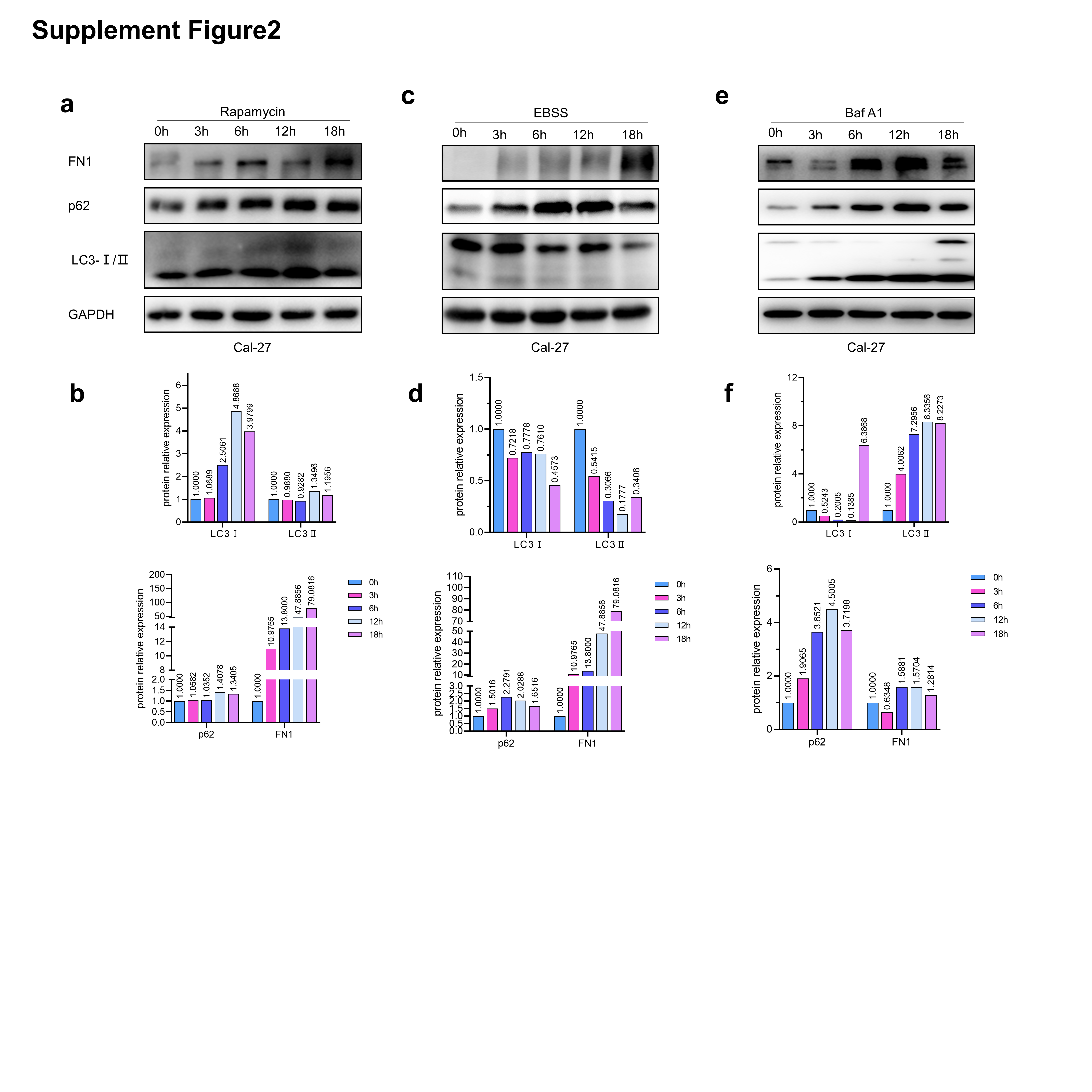

Supplement: Supplementary file 2 — Supplement Figure 2 [file 41368_2020_101_MOESM2_ESM.tif]

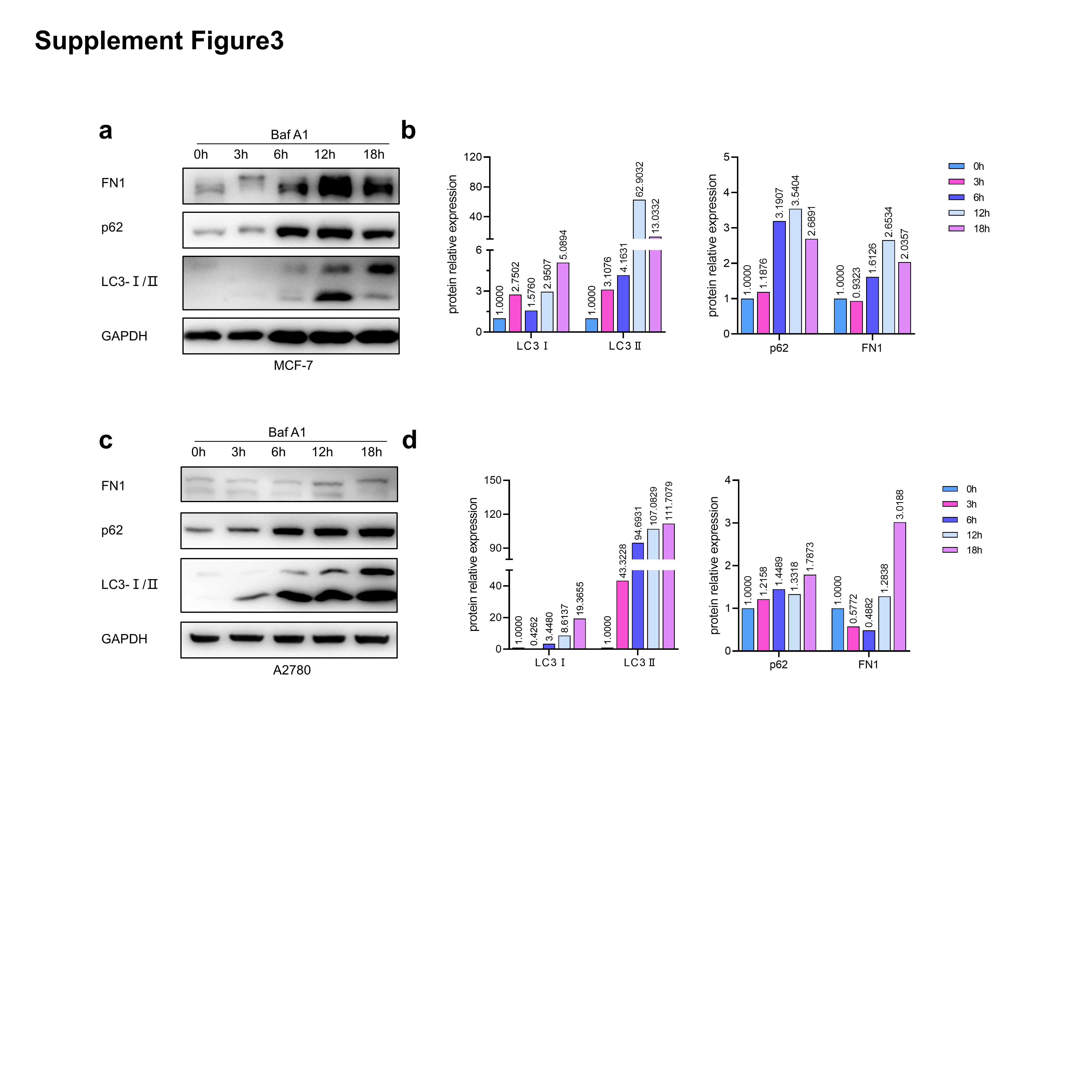

Supplement: Supplementary file 3 — Supplement Figure 3 [file 41368_2020_101_MOESM3_ESM.tif]

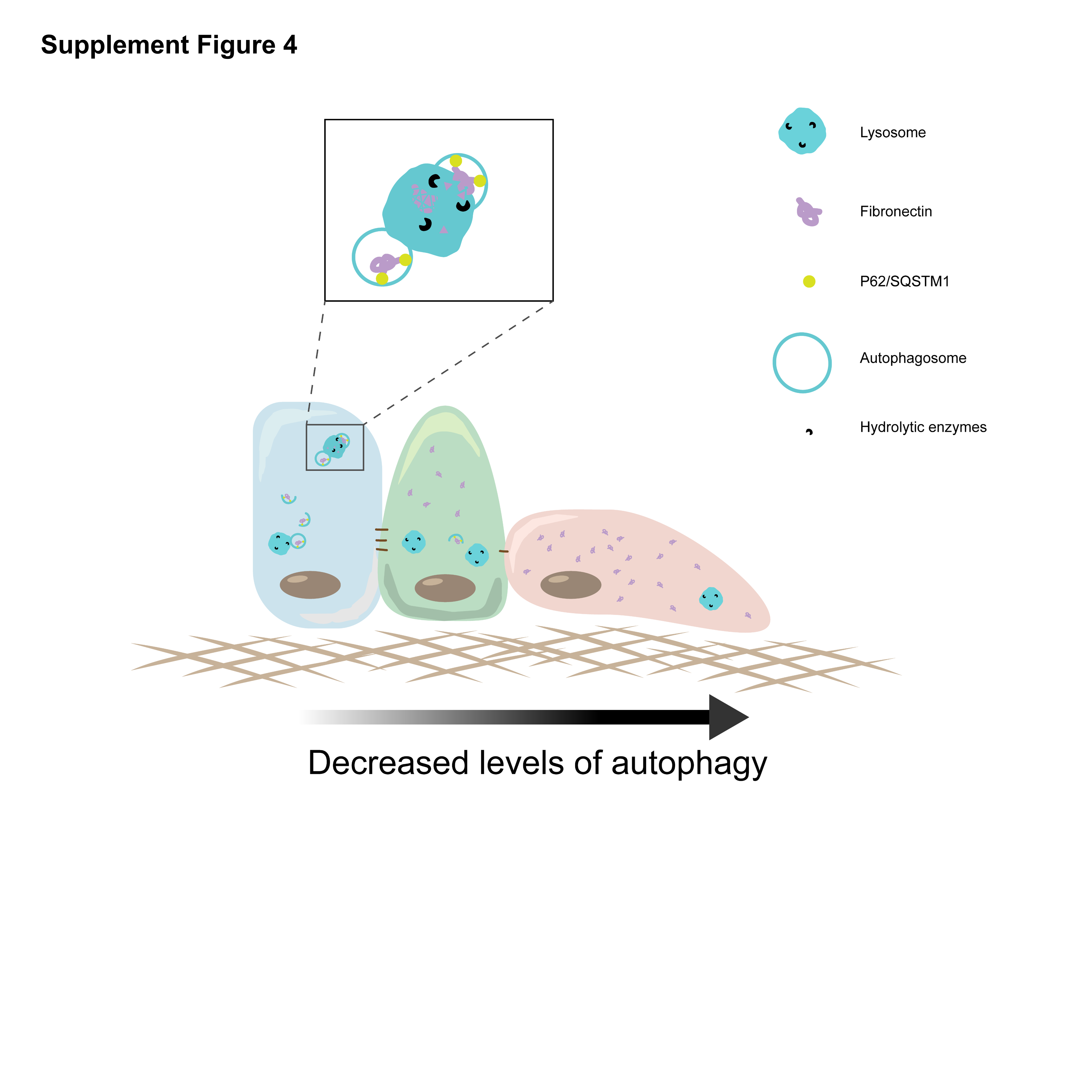

Supplement: Supplementary file 4 — Supplement Figure 4 [file 41368_2020_101_MOESM4_ESM.tif]

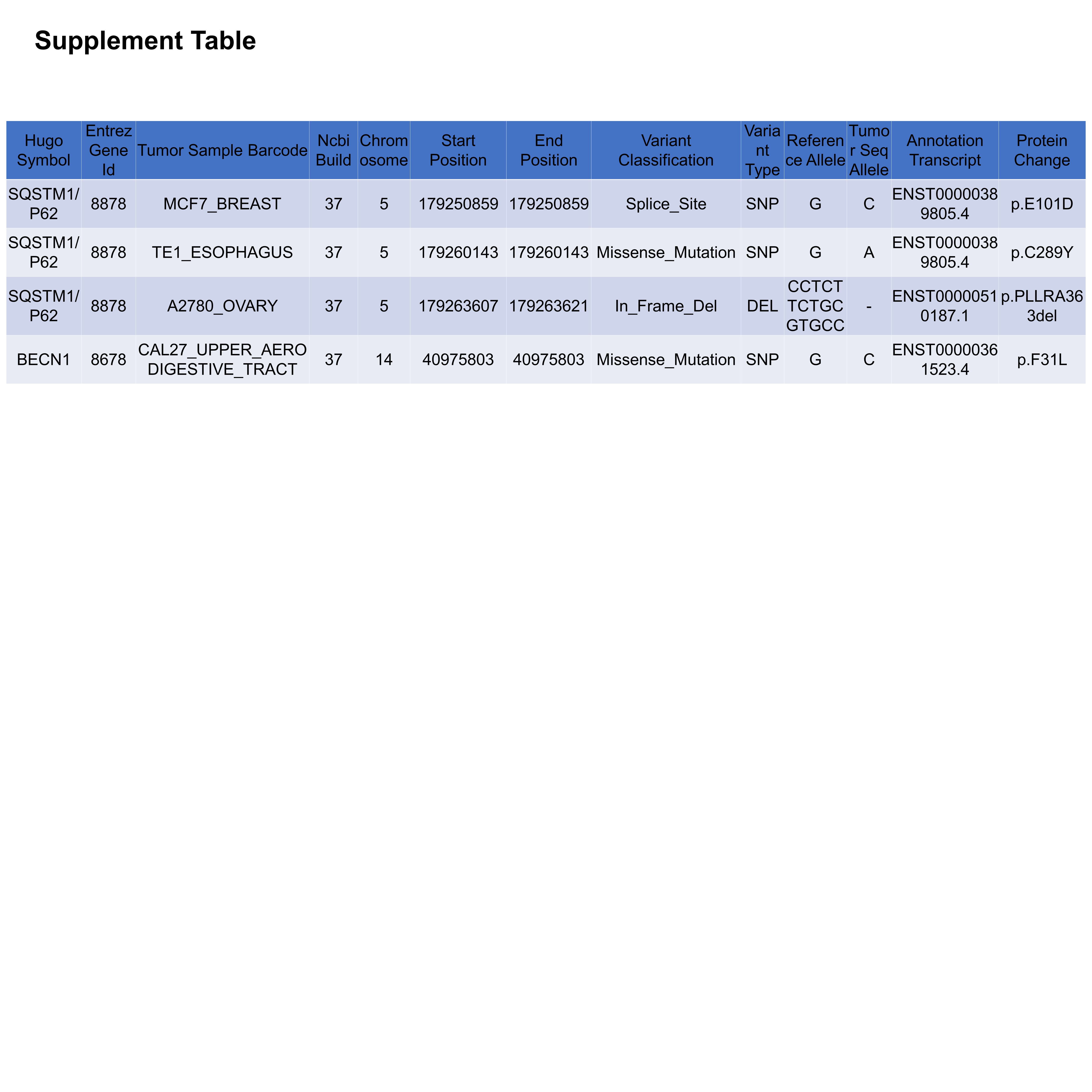

Supplement: Supplementary file 5 — Supplement Table [file 41368_2020_101_MOESM5_ESM.tif]
